# Regulatory requirements and labeling of commercially available prescription (oral) medicines in Sri Lanka: there is room for improvement

**DOI:** 10.1186/s40545-022-00409-z

**Published:** 2022-03-01

**Authors:** Manori Jayasinghe, Thotawaththage Loshadhi Indunika Srilal, Deweni Guruge Pathmila Prasadi, Wickramasinghe Senanayakege Sachini Madushika, Samanda Marakkala Dileka Udyani Silva, Sewwandi Subasinghe

**Affiliations:** Department of Pharmacy, Faculty of Allied Health Sciences, University of Ruhuna, Matara, Sri Lanka

**Keywords:** Policy, Labeling of drugs, Regulations, National Medicines Regulatory Authority, NMRA, Medication errors, Safe use of medicines, Medicine label information, Medicine packaging

## Abstract

**Background:**

The consistency and the quality of medicine labels are sought through the regulatory frameworks. This study aims at investigating the secondary labels of medicines based on the labeling regulations and guidelines issued by the National Medicines Regulatory Authority (NMRA), Sri Lanka.

**Methods:**

A descriptive cross-sectional study was conducted on 53 commonly used prescription-only oral medicines selected using the price regulations published for most commonly used drugs. High-resolution images of 216 brands/branded generics/generic products’ secondary labels were collected in April 2021 from six community pharmacies in six districts chosen as a convenience sample. Each label was manually assessed using a checklist prepared based on the regulatory requirements by four trained investigators. The status of registration of each product was assessed using the NMRA website. Descriptive statistics were performed.

**Results:**

There was a variation observed in labeling regulations and information present on packages. Among the 216 products evaluated, only 148 (68%) products appeared as registered medicines on the NMRA website, and 2.3% of medicines fulfilled all stipulated labeling parameters set out by the NMRA, 3% of products abided by the general labeling requirements, and 76% of the products complied with labeling requirements for API. Major deficiencies were observed in the presentation of registration numbers and the details of the local agent, which were unaccounted for in 210 (97%) and 131 (61%) products, respectively. The highest consistency (100%) of information was noted with the dosage form, date of manufacture, date of expiry, and batch numbers. Among the restricted information, attractive pictures (2%), web addresses (6%), and over-stickers (34%) were found.

**Conclusions:**

The results highlighted a gap between regulatory requirements and practice in medicine labeling information. Regular post-market examination of medicinal labels is highly advised in a country that relies largely on imports. Similarly, careful adherence to the labeling regulations is required. Furthermore, suppliers and local agents should be held accountable for ensuring accurate medicine labeling through increased awareness, education, and sanctions.

## Background

The importance of proper labeling of medicines in minimizing medication errors are well known [1]. Falsified, misleading, unclear, insufficient, and ambiguous information given on labels is among the major defects reported to be associated with improper labeling practices [2–5]. Thus, enhancement of the consistency and the quality of medicine labels are sought through the regulatory frameworks.

Labeling regulations in Sri Lanka aims at facilitating the proper identification of the product, mitigating medication errors, improving traceability, and achieving optimal therapeutic outcomes. According to the National Medicines Regulatory Authority (NMRA) of Sri Lanka, labeling includes “any printed, stenciled marked, embossed, or impressed text or graphic matter on the immediate container, on the outer pack, and any other printed material supplied together with the medicinal product” [6]. The NMRA has published the labeling guidelines to be implemented with medicines regulations ‘Part VIII’ of National Medicines (registration and licensing of medicine) Regulations, 2019 [6, 7]. These directives are expected to contribute towards proper handling and storage of the medicines [6].

The labeling of medicines in Sri Lanka is controlled at several stages as given below [6].

1. Primary label; label on the primary container.
2. Secondary label; label on the outer packaging of the container holding the primary pack.
3. Dispensing label: a label issued at the point of dispensing to users giving specific instructions.
4. Product information leaflet/package insert (PI).
5. Patient information leaflet (PIL).
6. Summary of Product Characteristics (SmPC).

During the process of market authorization, the NMRA evaluates the primary, secondary labels, PI, PIL, and SmPC. If inadequacies are found, regulators request to correct them before awarding the registration. Upon approval of the labels, it is the responsibility of the market authorization holders (licensed importers) in Sri Lanka for imported products to market the products using the approved labels. The same should be ensured by the domestic drug manufacturers themselves. In this study, we focused on the secondary labels on the outer packaging as we observed non-compliance with label requirements during community pharmacy visits. Postmarked secondary labels of the most used commercially available oral prescription medicines were assessed to check compliance with the labeling regulations and guidelines issued by the NMRA, Sri Lanka.

## Methods

A descriptive cross-sectional study was conducted on the commonly used medicines in Sri Lanka. The pricing regulation [8] published for commonly used medicines in 2019 was used to select medicines for the study as it is the most recently published reliable source of commonly used medicines published by the NMRA. Among the 60 medicines listed in the pricing regulation, 53 prescription-only oral dosage forms were selected for the study by excluding the over-the-counter medicines (*n* = 01), injections (*n* = 04), and inhalation dosage forms (*n* = 02). This study was confined to prescription-only oral medicines as there are additional labeling requirements applicable to over-the-counter medicines, injections, and inhalation products. When there were several strengths available for a medicine the highest strength was included in the sample. For the selected 53 medicines, high-resolution images of the secondary labels of all commercially available brands/branded generics/generic products were collected from six community pharmacies in six administrative districts of Sri Lanka selected as a convenience sample. Images were collected from 1st April 2021 to 30th April 2021. During this study, only the secondary labels were assessed as the regulations and guidelines are slightly different for other types of labels. When the same image of the brand/branded generic/generic product label was collected from several sites, only one label was counted for the assessment.

A checklist was developed to assess the secondary labeling requirements for oral prescription medicines marketed in Sri Lanka against the National Medicines Regulatory Authority Act no. 5 of 2015 [9] and National Medicines (Registration and Licensing of Medicine) Regulations, 2019 [7]. Against each variable in the checklist, responses were given as “Yes” “No” or “Not applicable” to be selected by the label evaluators during the assessment. The response: “Yes” was chosen when the claim is relevant to the product and present in the label under evaluation, “No” was selected when the claim is relevant to the product under evaluation, but it was absent on the label, “Not applicable” was chosen when the claim is not relevant to the product under evaluation.

During the formation of the checklist following criteria were excluded due to lack of specificity of the claims needed for the assessment: legibility and display of information, brand name, QR codes/2D barcodes, excipient related criteria, foreign language texts present on the drug labels, and differentiation of strengths of the same product. Further, requirements for parenteral and sterile products, over-the-counter products, and inhalation products were excluded from the checklist.

The status of registration of each product was assessed using the NMRA website. Other special warnings and precautions that may be necessary for the medicinal products were assessed using the British National Formulary (BNF 80).

There were four trained regulatory assessors engaged in the label assessment. Each label was manually evaluated by two evaluators independently against the checklist developed. When the observations and conclusions made by two investigators were different for the same product, the evaluation of the label by the regulatory pharmacist/principal investigator (MJ) was taken for the analysis. Descriptive statistics were performed using Microsoft® excel® 365. The results were expressed as frequency and percentage against each variable in the checklist.

## Results

This study examined 216 product labels of 53 commonly used medicines available in community pharmacies in Sri Lanka. The distribution of evaluated medicines in different Anatomical Therapeutic Chemical (ATC) classifications is given in Table 1.

**Table 1:** The ATC classification of the products evaluated

ATC index		Number of medicines included in the study	Number of different product labels (brands/branded generics/generics) evaluated
N03	Antiepileptics	6	10
N05	Psycholeptics	3	7
N06	Psychoanaleptic	1	3
C05	Vasoprotectives	1	1
C07	Beta blocking agents	1	3
C08	Calcium channel blockers	2	6
C09	Agents acting on the renin–angiotensin system	4	26
C10	Serum lipid reducing agents	2	29
J01	Antibacterial for systemic use	10	29
J05	Antivirals	1	1
B01	Antithrombotic agents	2	15
M01	Anti-inflammatory and antirheumatic products	3	13
M05	Drugs for treatment of bone diseases	1	1
A02	Drugs for acid related disorders	4	14
A03	Drugs for functional gastrointestinal disorders	1	5
A10	Drugs used in diabetes	6	39
H02	Corticosteroids for systemic use	1	3
H03	Thyroid therapy	1	1
R03	Drugs for obstructive airway disease	1	6
G04	Urologicals	1	3
P02	Anthelmintics	1	1
Total		53	216

Among the 216 products evaluated, only 148 (68%) products appeared as registered medicines on the NMRA website by the 14th of June 2021 [10]. The official or generic name was expected to be written following the recommended format (i.e., Metformin Tablets B.P. 500 mg). However, only 142 (66%) products complied with this requirement. The breakdown of the criteriawise assessment is given in Table 2.

**Table 2:** Assessment of secondary labels of medicines against the criteria mandated by the National Medicines Regulatory Authority, Sri Lanka

Criteria to be displayed on the label	Yes	No	NA
General labeling requirements			
The official/generic name in the recommended format (e.g., metformin tablets B.P. 500 mg)	142 (66%)	74 (34%)	0
The registration number issued by the authority	6 (3%)	210 (97%)	0
Pack size: the number of doses, weight, or volume contained in the pack	212 (98%)	4 (2%)	0
The dosage form	216 (100%)	0	0
Storage temperature	211 (98%)	5 (2%)	0
The date of manufacture in clear terms (month/year)	216 (100%)	0	0
The date of expiry in clear terms (month/year)	216 (100%)	0	0
The batch or lot number	216 (100%)	0	0
The name of the manufacturer and address of the manufacturing site	214 (99%)	2 (1%)	0
The name and address of the local agent	85 (39%)	131 (61%)	0
A special warning to store the product out of reach of children	187 (87%)	29 (13%)	0
General criteria restricted on secondary labels			
The availability of attractive pictures	4 (2%)	212 (98%)	0
Web addresses	12 (6%)	204 (94%)	0
Use of over stickers	74 (34%)	142 (66%)	0
Active pharmaceutical ingredient related labeling criteria			
The active ingredient is given by its International Nonproprietary Names (INN)	205 (95%)	11 (5%)	0
The quantity of each active ingredient expressed in terms of weight, volume, capacity, or units of activity	209 (97%)	7 (3%)	0
The active substances present as salts are indicated	119 (55%)	52 (24%)	45 (21%)
Product-specific labeling criteria			
Conform with Pharmacopeial labeling requirements	35 (16%)	8 (4%)	181 (80%)
Any other special warnings and precautions that may be necessary for the medicinal product	1 (0.46%)	3 (1.39%)	215 (98%)
Specific instructions if the product needs to be reconstituted, diluted, or prepared by any other means prior to its use	4 (2%)	0	212 (98%)
The period for which the medicine can be used after opening of the container or after reconstitution of the product	4 (2%)	0	212 (98%)

Yes—the claim is relevant to the product and present in the label under evaluation, No—the claim is relevant to the product under evaluation, but it was absent on the label, Not applicable/NA—the claim is not relevant to the product under evaluation

The medicine registration number was missing in 210 (97%) of the labels. However, most (94%) of the labels presented with several other numbers (e.g., SR No., CDDA Reg. No., FR No., numbers without reference), which were not the medicine registration number issued by the local authority. Several labels (3%) provided a medicine registration number issued by the Sri Lankan authority, which did not match with the registration number appearing on the NMRA website. Three products (0.4%) indicated registration numbers issued by the former Cosmetics devices and drugs authority (CDDA). In 3 (0.4%) labels, the registration numbers presented under the title of “Local registration no” were issued by other countries (e.g., Myn reg no., MM reg no.).

The pack size (a statement of net content), dosage form, date of expiry, and batch numbers were found in all the labels (100%). However, the presentation of information was different. Pack sizes were displayed following various formats (e.g., 100 tablets/10 × 10/3 blisters of 10 tablets/5 blisters @ 10 fast-melting tablets). The dates of manufacturing and expiry were expressed following several different methods (e.g., 1122, May 2020, 05/20, 05.20).

The specific storage temperature was given in 211 (98%) products. However, the method of writing storage information was observed to be different (e.g., Store in shade away from heat and moisture, store at a temperature not exceeding 25 °C, store below 25 °C, store up to 30 °C, do not store above 30 °C). It was noted that the information on storage, warning, and special instructions was given just as a passage making it difficult to quickly locate the information.

Attractive pictures were present in 4 (2%) of the labels. However, many labels contained different color lines and geometric shapes which were eye-catching, but difficult to categorize as definitive pictures. Further, in some labels, the key information was covered by externally affixed labels (34%) such as price tags and stickers with the name of the market authorization holder.

Requirements for the inclusion of the active pharmaceutical ingredient’s (API) details on the labels are given clearly in the regulations. Most of the products mentioned the name of the active ingredient using its INN 205 (95%), in terms of weight per dosage unit 209 (97%). The active ingredients present as salts were expressed as per the requirements given by the NMRA “e.g., each capsule contains Amoxicillin trihydrate equivalent to Amoxicillin 250 mg” only in 119 (55%) of the evaluated products.

As shown in Table 3, only 2.3% of medicines fulfilled all stipulated labeling parameters set out by the NMRA, 3% of products abide by the general labeling requirements, and 76% of the products complied with labeling requirements for API.

**Table 3:** Total number of products complied with all labeling criteria, general labeling requirements, and API labeling conditions of secondary labels

Labeling information	Number of products (*n* = 216)
Comply with all labeling requirements	5 (2.3%)
Comply with general labeling requirements listed in Table 2	6 (3%)
Comply will all labeling requirements for API	164 (76%)

## Discussion

According to the Central bank, in the year 2019 Sri Lanka had imported USD 553.13 million (LKR 98.82 billion: average exchange rate in 2019 was taken as 178.6566 LKR) worth medical and pharmaceutical products [11]. Due to high amounts of imports, there is a variation observed in terms of the information, format, and design of product labels. Thus, for all the products that are in the market, NMRA mandates “draft artworks, specimens, or mock-ups of outer cartons and primary labels submitted in the dossier to be consistent with the formats, designs, and colors of the original labels that would be used on commercial packs to be marketed” [6, 7]. In practice, it was observed that the information given on product labels tends to be falsified, misleading, unclear, insufficient, or ambiguous. To our knowledge, there were no published records, that assess the variability of information on secondary drug labels in the Sri Lankan market. Thus, this study provides important insights into the regulatory policy and practice.

The study found that there is a high variation in secondary labeling of medicines and information presented which challenge safe medication use. Among the 216 products evaluated, only 68% of products appeared as registered medicines on the NMRA website. Only 2.3% of the products evaluated complied with all the key labeling criteria and 3% of the products with general labeling criteria set by the NMRA. These findings highlight the importance of post-marketing surveillance of product labels to ensure that the approved labels are being used in product commercialization. User testing to guarantee optimum clarity of key information is desirable and regarded as a best practice around the world [12].

Provision of the name and address of the local agent is expected by the regulations to assist in the quick identification of the company responsible for marketing the product. Abiding regulatory requirements of imported medicines are a shared responsibility of manufacturers and local market authorization holders approved by the NMRA. Regulatory pharmacists’ awareness of the importance of proper labeling and jurisdictional requirements can enhance the accountability in commercializing medicines with approved labels. Further, NMRA initiated post-marketing surveillance, and penalties on inadequate or falsified labeling could heighten the legal responsibility of the marketers.

The uniform writing style of the generic name can help in documentation, international trade, and medication dispensing. Following common names (e.g., atorvastatin calcium tablets and atorvastatin tablets) by different suppliers not indicating the strength in the generic name can be deleterious when multi-strength products are available. Enforcing regulations to unify the presentation of generic names on labels is advisable.

Further, proper labeling can assist in controlling the availability of falsified medicines in the market. Confronting this challenge NMRA has mandated printing the Medicine Registration Number issued by NMRA on the drug labels, facilitating the easy identification of registered products by cross comparing it with the registration number given on the NMRA website. This was not a requirement under the former Cosmetics Devices & Drugs Act no. 27 of 1980 and its regulations [13]. However, the findings suggest that the marketers are not following this rule. Thus, it will be highly efficient to develop a mechanism to check the registration number printed on the packages at the point of custom clearances to prevent the entry of falsified products to the country. The easy identification of the registration number from the rest of the label content is important. The presence of multiple meaningless numbers was found to be overcrowding the labels hindering the noticeability of critical information. Investigators find the European union’s guidelines suggesting the use of a blue-colored box, where marketers need to display the product registration number to be useful in addressing this issue [14].

Furthermore, Sri Lanka is categorized under Zone IVb, and medicines are expected to test for stability at 30 °C/75% relative humidity levels [15]. Consequently, the details of the shelf-life and storage information are expected to be provided accordingly. However, most drug labels instruct to store at or below 25 °C. Achieving this temperature is only a possibility until the drugs are dispensed from pharmacies. However, as the household temperature exceeds 25 °C, people tend to refrigerate products to achieve ‘cool’ conditions. Many people store medicines under normal environmental conditions and ultimately consume deteriorated/substandard products. Therefore, regulations should be updated to impose stability testing of medicines based on real environmental conditions.

Looking at the criteria that are prohibited on the labels: First attractive pictures are not allowed based on the principle of reducing the attractiveness of medicines to children [6]. Even though pictures were absent in the test sample, the variation of colors, mixing of color lines create attractiveness to the package. These variations help in the easy identification of different strengths of the same products or different products manufactured by the same manufacturer [16]. Second, the indication of company websites, promotional internet websites, or other information sources is forbidden. However, product information websites can be a source of information to help proper medication use. Third, prevention of the use of over stickers is important to protect the integrity of original labeling information. This is only permitted to display retail price and details of the local company.

## Limitations

Though the NMRA website is a reliable source of information less frequent updates might have interfered with the registration information of the products evaluated in this study. Further, the inability to cross-compare and assess the information about API, details of the manufacturers, batch releasers, and outsourced activities submitted in the registration dossier was a limitation of this study. Further, the generalization of product-specific labeling information was not successful due to the small number of medicines in the sample required to have product-specific labeling information. Therefore, additional investigations on these parameters are essential.

## Conclusions

Findings of the assessment of secondary labels of medicines highlighted a gap between regulatory requirements and practice. As a country depending heavily on importations, the need for regular post-market assessment of medicine labels is highly recommended. Similarly, strict enforcement of the labeling guidelines is a necessity. Additionally, the accountability of suppliers and local agents in ensuring proper drug labels should be attained through raising awareness, education, and sanctions.

## Data Availability

The datasets generated and/or analyzed during the current study are not publicly available due to tradenames present in it but are available from the corresponding author on reasonable request.

## Abbreviations

NMRA: National medicines regulatory authority
ATC: Anatomical therapeutic chemical
CDDA: Cosmetics devices and drugs authority
API: Active pharmaceutical ingredient
INN: International nonproprietary names
API: Active pharmaceutical ingredient
PI: Product information leaflet/package insert
PIL: Patient information leaflet
SmPC: Summary of Product Characteristics
